# miRNA Expression Profile in Whole Blood of Healthy Volunteers and Moderate Beer Consumption with Meals

**DOI:** 10.3390/nu18010149

**Published:** 2026-01-01

**Authors:** Teresa Padro, Rafael Escate, Lina Badimon

**Affiliations:** 1 Institut Recerca Sant Pau (IR Sant Pau), Sant Quintí, 77-79, 08041 Barcelona, Spain; rescate@santpau.cat (R.E.); lbadimon@ficsi.org (L.B.); 2 Centro de Investigación Biomédica en Red Cardiovascular (CIBERCV), Instituto de Salud Carlos III, 28029 Madrid, Spain; 3 Medical School, Universitat de VIC-UCC and IRIS-CC, 08500 Vic, Barcelona, Spain; 4 Cardiovascular Research Foundation for Health Prevention and Innovation (FICSI), 08017 Barcelona, Spain

**Keywords:** miRNAs, fermented beverages, beer, inflammation signaling, in silico analysis, cytokines

## Abstract

**Background/Objectives:** Moderate consumption of fermented beverages such as traditional beer has been associated with antioxidant and anti-inflammatory effects. MicroRNAs (miRNAs) play key roles in inflammation and oxidative stress, yet the impact of moderate fermented beverage consumption on blood miRNA profiles remains poorly understood. This study investigated the effects of regular, moderate intake of traditional and alcohol-free beer on whole blood miRNA levels in healthy adults. **Methods:** Whole blood samples were collected at baseline and after a 4-week intervention with alcohol-free beer and traditional beer in healthy overweight/obese adults (*n* = 36). miRNA profiling was performed using Affymetrix in a discovery subset, followed by targeted validation using real-time PCR in the full cohort. Bioinformatics and system biology analysis were applied to explore potential functional associations. **Results:** After traditional beer consumption, 202 miRNAs showed differential expression compared to baseline (*p* < 0.05). Eighteen miRNAs with changes ≥1.5-fold and the two miRNAs with the lowest *p*-values (*p* < 0.005) were selected for further analysis. Of them, the six miRNAs with the most consistent expression patterns were validated by real-time PCR. Moderate beer intake was associated with increased levels of miR-144-5p and miR-19a-3p in the overall population. Sex-stratified analyses suggested a tendency toward higher levels in these miRNAs in women following traditional beer intake. In silico analysis showed that predicted target genes of these miRNAs are involved in pathways related to immune regulation and inflammatory signaling. **Conclusions:** Moderate beer consumption is associated with consistent changes in whole-blood miRNA expression, particularly miR-144-5p and miR-19a-3p, in a healthy overweight/obese population. These findings support a potential role for epigenetic modulation in the biological response to moderate beer intake and provide a basis for future mechanistic studies.

## 1. Introduction

Lifestyle and diet are major determinants in the development of non-communicable diseases (NCDs). A general consensus supports adherence to the Mediterranean diet for their prevention [1]. The essence of the Mediterranean diet not only considers the use of natural, whole foods and the avoidance of highly processed and pre-cooked foods but also low-to-moderate daily ingestion of fermented beverages [2,3].

Low-to-moderate consumption of fermented beverages such as wine or beer is related to health benefits and reduced presentation of cardiovascular diseases [4], while high levels of alcohol intake are associated with increased cardiovascular risk [5,6]. Interestingly, sex differences related to alcohol metabolism have not been studied in depth [7,8,9].

Fermented beverages have been suggested to exert their beneficial effects through their antioxidant properties [10] and the regulation of inflammasome pathways [11]; however, there is a need to investigate cellular and molecular mechanisms that scientifically demonstrate how fermented beverages affect the cardiovascular system.

Chronic inflammation is now recognized as an overwhelming burden for public health [12,13], contributing to aging and the pathogenesis of non-communicable diseases such as rheumatoid arthritis, diabetes, periodontitis, and atherosclerosis. In particular, the immune response and the inflammation play a central role in the initiation and progression of atherosclerosis, driving immune cell recruitment, cytokine production, and endothelial dysfunction, which collectively exacerbate plaque development and vulnerability [14,15]. Whether fermented beverages can modulate the innate immune response and how these tentative effects are produced is a matter of high interest.

Functional genomics provides a framework to explore dynamic regulatory processes influencing gene expression at transcriptional and post-transcriptional levels. Non-coding RNAs such as microRNAs (miRNAs), which are small RNAs (∼22 nucleotides in length), are key post-transcriptional regulators that control gene expression by promoting mRNA degradation or inhibiting translation [16]. Importantly, miRNA levels and activity can be modulated by environmental and dietary factors, including nutrients, food components, and bioactive compounds [17].

Beer, a plant-based fermented beverage, is rich in polyphenols and other bioactive components linked to beneficial effects on cardiovascular health [18]. We have previously demonstrated that serum from subjects drinking low–moderate amounts of beer could modulate the inflammasome in human macrophages from peripheral blood mononuclear cells (PBMCs) [11] and regulate the differentiated macrophage gene transcription [19], partially relating its effects to the polyphenolic component.

Based on these previous findings, here we investigated whether moderate and regular beer consumption could induce epigenetic regulation through the modulation of miRNAs. To this aim, we studied the differential miRNA profile in whole blood samples of healthy overweight/obese subjects after 4 weeks of regular and moderate intake of traditional and alcohol-free beer compared to baseline levels.

## 2. Materials and Methods

### 2.1. Study Population and Design

The study included 36 healthy adults with overweight (BMI 28–29.9 kg/m^2^, 53%) or class I obesity (BMI 30–35 kg/m^2^, 47%) to represent a metabolically stable population commonly exposed to moderate beer consumption and without additional cardiovascular risk factors. Participants were aged 40–60 years (100%), and 42% were women. Baseline clinical characteristics of participants are summarized in Table S1. Participants were enrolled in an open, randomized, two-arm, longitudinal cross-over trial comprising two 4-week intervention periods, separated by a 4-week wash-out, as previously described [10]. The present miRNA analysis represents an exploratory secondary analysis conducted within this prospective cross-over study. All were moderate beer consumers and completed a 4-week run-in period, abstaining from beer and other fermented or alcoholic beverages before starting the interventions.

During the study (run-in, wash-out periods, and intervention phases), participants refrained from consuming any alcoholic beverages or alcohol-free beer other than those provided in the study. During intervention phases, men received 600 mL/day (two cans) and women 300 mL/day (one can) of traditional or alcohol-free beer from the same Spanish commercial brand. Each can of alcohol-free beer contained 0.0 g alcohol and 414 mg polyphenols, while traditional beer provided 15 g ethanol (5.7% vol) and 604 mg polyphenols per can.

Dietary intake was assessed using food frequency questionnaires before each visit, with minimal reported changes. Adherence was monitored via regular phone contact, end-of-phase interviews, and daily beer intake logs. At the end of each phase, a clinician evaluated participants for potential adverse effects, including flushing, abdominal discomfort, dizziness, vomiting, and diarrhea. Biological samples were obtained at baseline and at the end of each intervention period, as previously described [10]. The study was conducted in accordance with the Declaration of Helsinki and approved by the Human Ethical Review Committee of the Hospital “Santa Creu i Sant Pau” of Barcelona, Spain (Ref. 14/186; 12 November 2014). All participants provided written informed consent prior to enrolment.

miRNA profiling was conducted using whole blood samples at baseline and after each intervention period for all participants. The study was performed in two sequential stages: a derivation stage, involving a high-throughput screening to identify differential miRNA expression at baseline and after the 4-week intervention with traditional beer (*n* = 10); and a validation stage, in which the selected miRNAs—identified through high-throughput—were quantified by real-time PCR in the full intervention cohort (*n* = 36) [10] at baseline and after the 4-week interventions with alcohol-free or traditional beer.

### 2.2. Sample Collection

Whole human blood samples were withdrawn from the cubital vein without a tourniquet using a 20-gauge needle after 10–14 h of fasting. A 2.5 mL aliquot of the sample was collected in PAXgene Blood RNA tubes, which capture both cellular and circulating RNA components and are appropriate for systemic RNA analyses. Total RNA was isolated using the PAXgene Blood miRNA Kit (PreAnalytiX, GmbH, Hombrechtikon, Switzerland), according to the manufacturer’s instructions. Additionally, EDTA-tubes were collected for hematological analysis, and plasma samples were obtained after centrifugation at 1800× *g* for 20 min. Aliquots were immediately snap-frozen in liquid nitrogen and stored at −80 °C until analysis [10]. All samples were processed within 2 h of collection under standardized conditions.

Total RNA, including microRNA, was used for subsequent microarray and PCR analyses, following the manufacturer’s protocol. Each sample was spiked with 25 fmol of synthetic cel-miR-39 for normalization. RNA purity and concentration were assessed using a NanoDrop ND-1000 spectrophotometer (NanoDrop Technologies, Wilmington, DE, USA). Aliquots of purified RNA were snap-frozen in liquid nitrogen and stored at −80 °C until analysis.

### 2.3. miRNA Profiling: Affymetrix miRNA Analysis

miRNA profiling was performed using the Affymetrix GeneChip™ miRNA 4.0 Array (Thermo Fisher Scientific, Waltham, MA, USA). The miRNA array contains 6631 probes for short non-coding RNAs, including 32 controls, 1996 with non-miRNA nomenclature, 2025 miRNAs with pre-mature forms, and 2578 miRNAs with mature forms. For each sample, 250 ng of total RNA were processed according to the manufacturer’s protocol using the FlashTag™ Biotin HSR RNA Labeling Kit (Thermo Fisher Scientific) and analyzed on the GeneChip™ Scanner 3000 7G System, which includes the Scanner 3000 7G, Fluidics Station 450, and Hybridization Oven 645.

Briefly, a poly-A tail was added to RNA molecules, followed by a ligation reaction to attach a biotin label. Each biotin-labeled RNA sample was hybridized to the GeneChip™ miRNA 4.0 array cartridge and detected using an avidin–streptavidin–phycoerythrin (PE) conjugate, which binds specifically to the biotin-labeled RNA. Hybridization was carried out at 48 °C with rotation at 60 rpm for 17 h. After hybridization, arrays were washed and stained using the FS450_002 fluidics script. The arrays were then scanned, and raw data were processed using the Transcriptome Analysis Console (TAC) software v4.0 (Thermo Fisher Scientific). Data summarization and normalization were performed using the RMA-DABG procedure.

Data quality was analyzed using Expression Console software v1.3.1.187 according to the manufacturer’s instructions. Briefly, the quality of miRNA signals was assessed using the RMA and DABG algorithms. Labelling and array processing were tested with the spike-in control probe sets (spike_in-control-2_st, spike_in-control-23_st, spike in-control-29_st, spike_in-control-31_st, and spike_in-control-36_st) to confirm the quality of poly(A) tailing, ligation, and the absence of RNases in the RNA samples. The quality of array hybridization, washing, staining, and scanning procedures was assessed using four different probe sets (AFFX-r2-Ec-c1-BioB-3_at, AFFX-r2-Ec-c1-BioC-3_at, AFFX-r2-Ec-c1-BioD-3_at, and AFFX-r2-Ec-c1-cre-3_at).

Whole blood miRNA data obtained by the Affymetrix microarrays were deposited in NCBI’s Gene Expression Omnibus and are accessible under the accession number GSE310134 (https://www.ncbi.nlm.nih.gov/geo/query/acc.cgi?acc=GSE310134, accessed on 15 September 2025).

### 2.4. miRNA Quantification: Real-Time PCR Analysis

miRNA quantification was performed by reverse transcription to cDNA using a universal reverse transcription methodology with the ‘Taqman Advanced miRNA’ technology (Thermo Fisher Scientific). cDNA quantification was carried out using TaqMan-based real-time quantitative PCR (Thermo Fisher Scientific), according to the manufacturer’s instructions for the selected human miRNAs.

Specific miRNAs listed in Table S2 were analyzed in duplicate. Cycle threshold (Ct) values of <32 were consistently accepted. Normalization of miRNA expression was performed using cel-miR-39 as an exogenous reference control and miR-16-5p as an endogenous miRNA. Normalized expression levels were calculated using the ΔCt method according to the equation 2^−(Ct[target] − Ct[reference])^ [20].

The stability and quality of miRNA amplification were assessed based on predefined quality criteria, including consistent detection across all samples, amplification score (Amp Score) > 1, Cq confidence score (Cq Conf) > 0.8, and absence of amplification in negative controls. cel-miR-39 and miR-16-5p met these criteria in 100% of the analyzed samples, supporting their suitability as normalization controls.

Data were quantitated and analyzed using SDS 2.4 and Expression Suite v1.1 software (Thermo Fisher Scientific). PCR efficiencies were calculated with calibration curves (dilutions 1:2) and an algorithm that includes slope, intercept value for the *Y*-axis, and R2 value.

### 2.5. In Silico: Bioinformatics and System Biology Analysis

Bioinformatic analyses were performed using several databases with different search algorithms, including miTED, miRWalk, and Cytoscape, ShinyGO, and Ingenuity Pathway Analysis (IPA), to analyze the selected miRNAs and their predicted target genes.

The tissue sources of the miRNAs were obtained from the microRNA Tissue Expression Database (miTED; https://dianalab.e-ce.uth.gr/mited/#/expressions, accessed on 18 September 2025), which estimates miRNA abundance by analyzing thousands of raw small RNA-Seq (sRNA-Seq) datasets.

Potential target gene candidates were identified using the miRWalk v3.0 search algorithm (http://mirwalk.umm.uni-heidelberg.de/, accessed on 25 September 2025), a public resource that predicts miRNA binding sites across complete gene sequences with high probability. The predicted target genes were then analyzed using STRING, a bioinformatics tool accessed via the Cytoscape platform to obtain protein-coding RNA (https://cytoscape.org/, accessed on 2 October 2025). Cytoscape v3.10.4 is a platform software that provides a basic set of functions for data integration through multiple applications and different database connections.

Functional enrichment analysis of target genes was performed using ShinyGO v0.85 (http://bioinformatics.sdstate.edu/go/, accessed on 15 October 2025), focusing on KEGG and PANTHER signaling pathways. Analyses were based on Ensembl gene ID and STRING-db protein IDs. Functional enrichment was assessed using the hypergeometric test, with false discovery rate (FDR) correction using the Benjamin–Hochberg method, and fold enrichment was used to quantify the magnitude of gene representation.

IPA v153384343 (https://digitalinsights.qiagen.com/products-overview/discovery-insights-portfolio/analysis-and-visualization/qiagen-ipa/, accessed on 20 October 2025) was used to identify target genes related to inflammasome pathways. IPA is based on the IPA Knowledge Base, a curated repository of biological interactions and functional annotations derived from the published literature.

### 2.6. Statistical Analysis

Continuous variables are presented as medians with interquartile ranges, unless otherwise specified, while categorical data are expressed as absolute counts (*n*) and percentages. The distribution of continuous variables was assessed using the Kolmogorov–Smirnov test, and the homogeneity of variances in miRNA levels among healthy subjects was evaluated using Levene’s test. For all analyzed variables, a paired before–after design was used to maximize sensitivity to within-subject changes induced by the intervention. Comparisons between groups were performed using either the paired Student’s t-test or the non-parametric paired-Wilcoxon test. Correlations between continuous variables were determined using Spearman’s correlation and hierarchical clustering was conducted with Chiplot (https://www.chiplot.online/, accessed on 7 October 2025). Given the exploratory nature and the limited sample size of the discovery subset (*n* = 10) for the Affymetrix microarray analysis, nominal *p*-values from paired comparisons were used for candidate miRNA selection without false discovery rate (FDR) correction. Statistical analyses were performed using SPSS Statistics version 25 (IBM Corp., Armonk, NY, USA), R software 2025.09.2+418 (Posit Software, Boston, MA, USA), and StatView v5.0.1 (SAS Institute, Cary, NC, USA). A *p*-value lower than 0.05 was considered indicative of statistical significance.

## 3. Results

### 3.1. Differential miRNAs in Whole Blood of Healthy Subjects After Consuming Traditional Beer by Untargeted Affymetrix Analysis

A high-throughput untargeted analysis of miRNA profiles was performed using the Affymetrix platform on RNA samples obtained from whole blood of healthy subjects after a 4-week intervention with traditional beer, compared to their baseline samples collected before beer consumption (*n* = 10).

**Figure 1:**
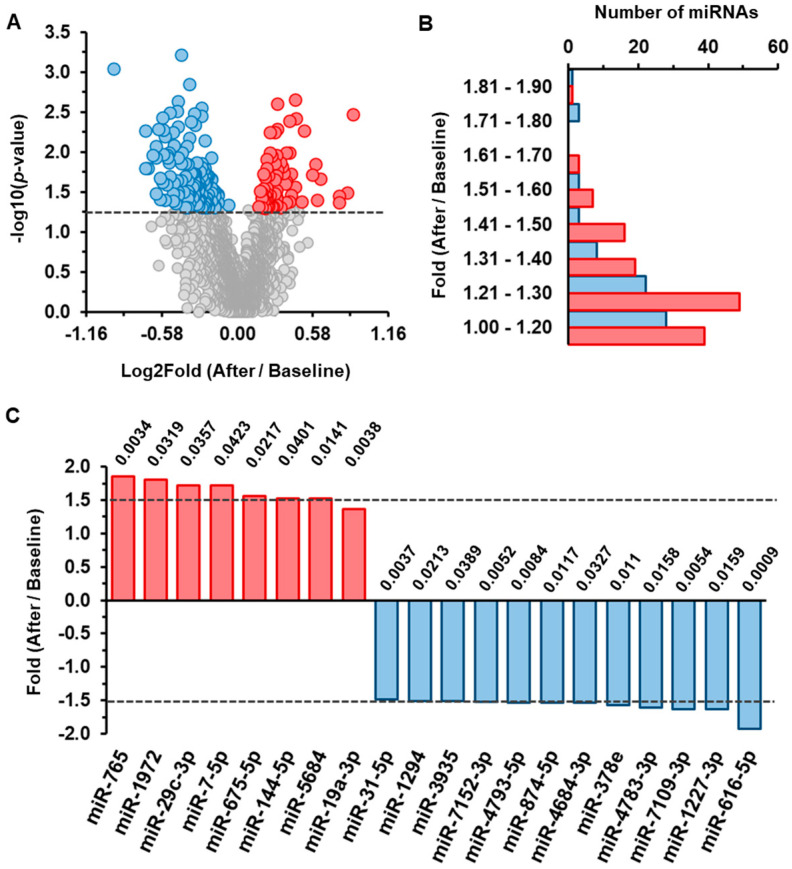
microRNA (miRNA) profiling by Affymetrix in healthy subjects after traditional beer. Whole blood miRNAs were analyzed by microarray (after vs. baseline, *n* = 10). (**A**) Volcano plot refers to fold change (*X*-axis) and Student’s *t*-test *p*-values (−log10, *Y*-axis). Red dots: upregulated miRNAs. Blue dots: downregulated miRNAs. (**B**) Ranges of fold changes for upregulated and downregulated miRNAs are shown in red and blue bars. (**C**) miRNAs with significant differential changes ≥ 1.5-fold (dotted line threshold) in response to traditional alcoholic beer are shown. *p*-values are indicated.

The analysis revealed 202 mature miRNAs with statistically significant differential changes (*p* < 0.05), of which 68 miRNAs were upregulated and 134 were downregulated (Figure 1A,B). Among these, a subset of 18 miRNAs exhibited fold changes of >1.5, which included 7 upregulated miRNAs and 11 downregulated miRNAs after beer consumption. These were selected for further analysis. miR-19a-3p and miR-31-5p, with the lowest *p*-values for change (*p* < 0.005), were also considered for the subsequent validation studies (Figure 1C).

### 3.2. Quantitative Real-Time PCR Analysis of Whole Blood miRNA Expression in Healthy Subjects After 4-Week Intervention with Alcohol-Free and Traditional Beer

Quantitative expression levels of miRNAs were analyzed by real-time PCR in the full study population (*n* = 36) at baseline and after the 4-week intervention with alcohol-free beer and traditional beer.

Among the 20 selected miRNAs, six showed consistently detectable expression across the study group, meeting quality criteria that included an amplification score > 1, a Cq confidence score > 0.8, cycle threshold (Ct) values < 32 in 100% of analyzed samples (Figure S1A–C), and non-detection of amplification in negative controls.

**Figure 2:**
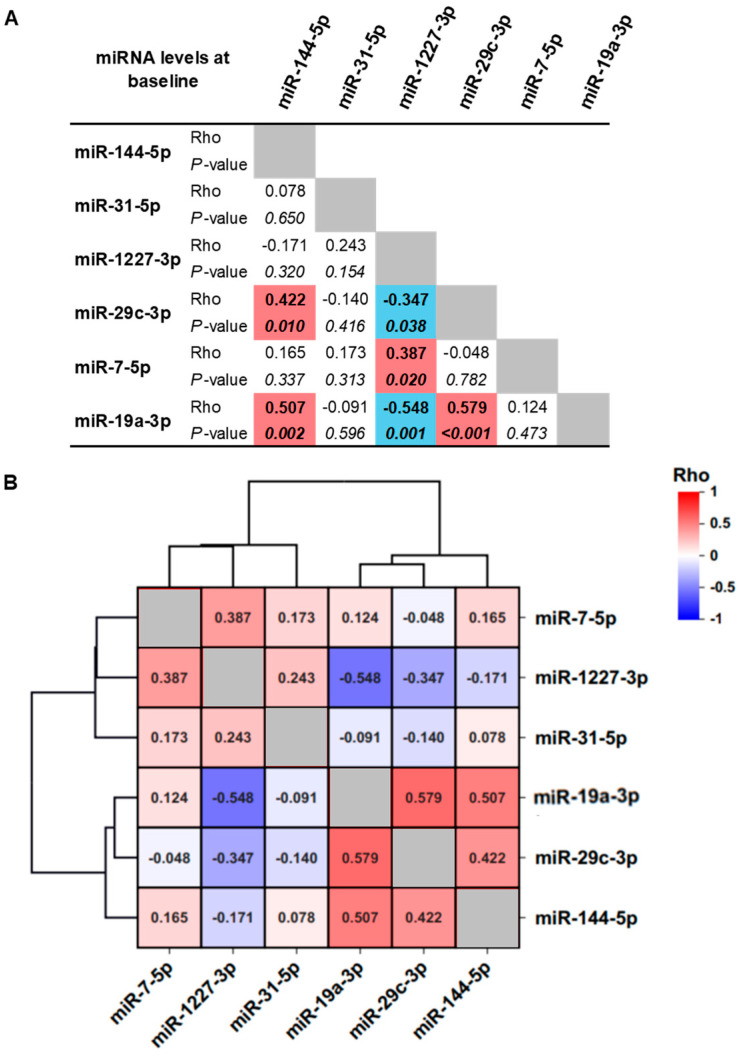
Correlations of miRNA levels in total population before drinking beer. Expression levels of miRNAs were analyzed by real-time PCR. (**A**) The correlations between miRNAs were performed by Spearman’s correlation. Positive correlations in red color. Negative correlations in blue color. Statistical correlations with *p*-values < 0.05 are shown in italic and bold. (**B**) Hierarchical clustering for Spearman’s correlation was performed. miR-144-5p, miR-19a-3p, and miR-29c-3p are the combination (cluster) with the highest level of hierarchy.

Spearman’s correlation analysis of these miRNAs revealed two distinct clusters based on significant associations in their baseline expression patterns: Cluster 1 comprising miR-144-5p, miR-29c-3p, and miR-19a-3p (positive correlations); and Cluster 2 comprising miR-1227-3p, miR-29c-3p, and miR-19a-3p (negative correlations). In addition, miR-1227-3p showed a positive correlation with miR-7-5p (Figure 2A). Hierarchical clustering (Ward’s method, Euclidean distance, and no standardization) further confirmed that the miRNAs in Cluster 1 (miR-144-5p, miR-29c-3p, miR-19a-3p) showed the highest similarity in expression patterns (Figure 2B).

**Figure 3:**
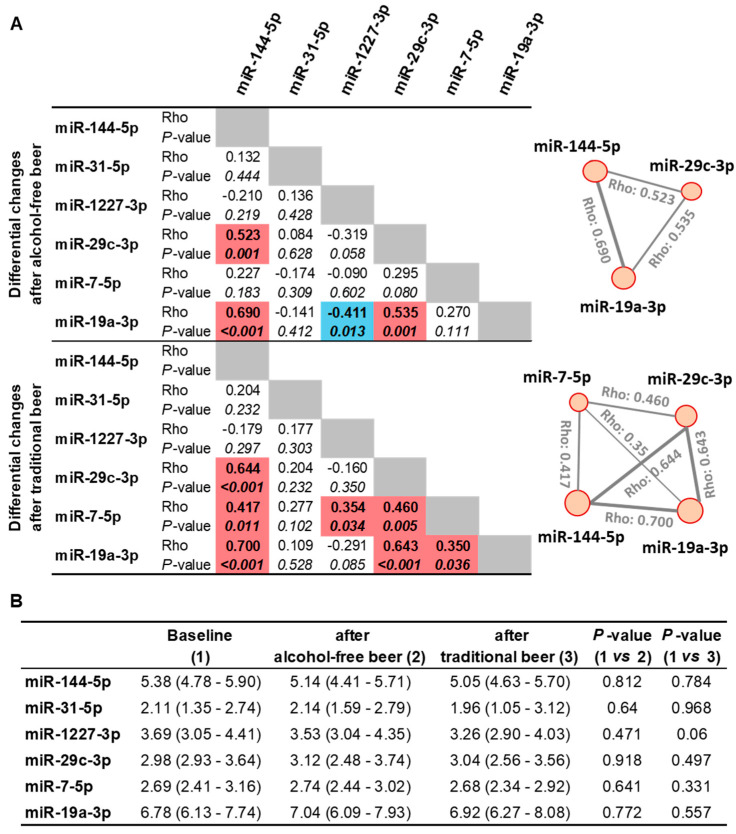
Expression levels of miRNAs at baseline and after beer consumption. (**A**) Spearman’s correlation between differential changes in miRNA levels (after–baseline). Positive correlations in red color. Negative correlations in blue color. Statistical correlations with *p*-values < 0.05 are shown in italic and bold. (**B**) miRNA levels after consumption of alcohol-free beer and traditional beer compared to baseline status before drinking beer. Results are shown as medians (Interquartile range, IQR: Q1–Q3). Statistical significance was calculated by a paired Wilcoxon test.

miRNAs in Cluster 1 also showed strong association patterns in their expression changes after the 4-week interventions compared to baseline, regardless of whether participants consumed traditional or alcohol-free beer (Figure 3A). Notably, miR-7-5p and Cluster 1 showed changes following the traditional beer intervention, although with a weaker association strength after alcohol-free beer. After beer consumption, miR-31-5p did not cluster with any other miRNA.

However, individual miRNA analyses revealed no significant differences in expression levels after the 4-week interventions with traditional or alcohol-free beer compared to baseline status for any of the six miRNAs studied (Figure 3B).

### 3.3. Differential Levels of miRNA Associated with Sex in Healthy Subjects After 4-Week Intervention with Alcohol-Free Beer and Traditional Beer

**Figure 4:**
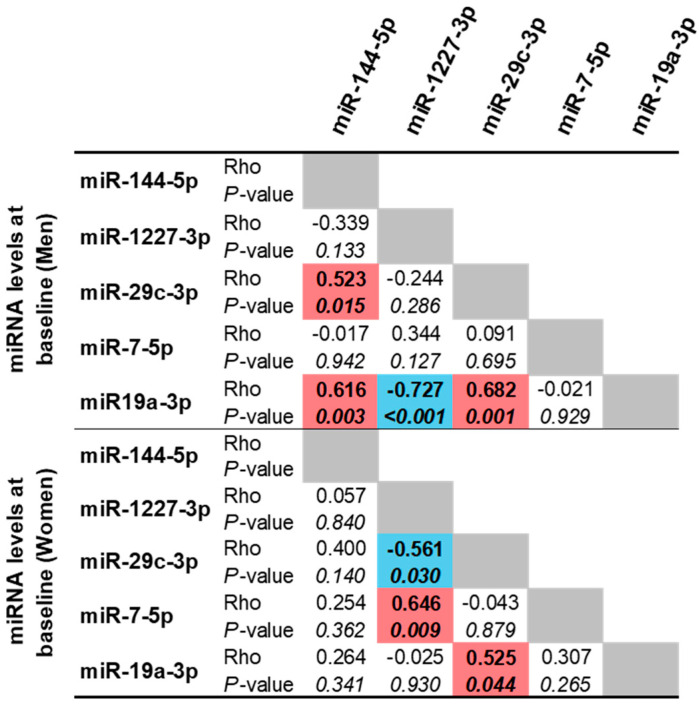
Correlations of miRNA levels before drinking beer in men and women. The correlations between miRNA levels at baseline were analyzed by Spearman’s correlation. Positive correlations in red color. Negative correlations in blue color. Statistical correlations with *p*-values < 0.05 are shown in italic and bold.

To investigate sex-related differences in miRNA levels following the consumption of both types of beer, we first analyzed baseline miRNA correlation patterns separately in men and women. As shown in Figure 4, the miRNAs in Cluster 1 showed significant associations in men. In women, these correlations were weaker for miR-144-5p with miR-29c-3p and for miR-144-5p with miR-19a-3p but remained significant between miR-29c-3p and miR-19a-3p.

**Figure 5:**
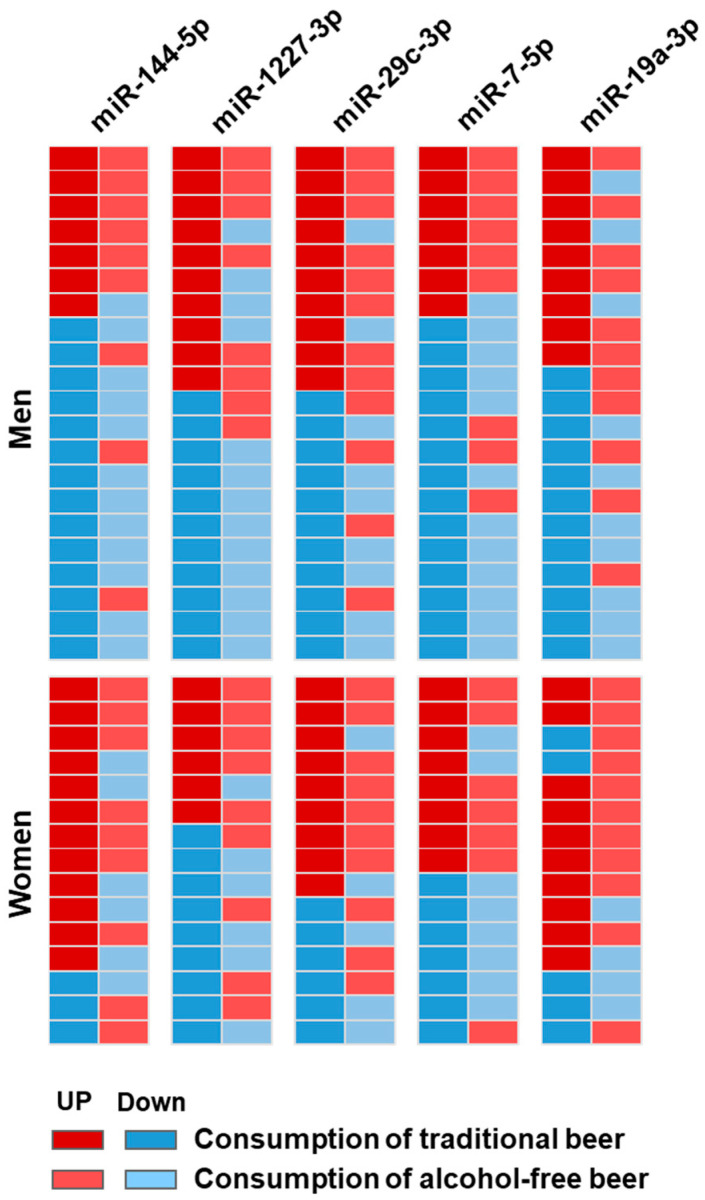
Differential changes after beer consumption in men and women. Differential changes in the miRNAs (after beer consumption compared to baseline) were calculated for men and women. Results are displayed as a heatmap; dark red and blue colors indicate higher and lower levels of the miRNAs found after traditional beer; light red and blue colors indicate higher and lower levels of the miRNAs found after alcohol-free beer.

When examining individual responses to alcohol-free and traditional beer using heatmap analysis (Figure 5), inter-individual variability in miRNA expression was observed. Men showed a greater tendency toward decreased expression of the five selected miRNAs, whereas women exhibited a tendency toward increased expression after the intervention period. This sex-related pattern was more apparent for miR-144-5p (men: decreased expression in 66.6% of participants; women: increased expression in 88%; Chi-square < 0.05) and for miR-19a-3p (men: decreased expression in 61.9%; women: increased expression in 88%; Chi-square < 0.05). These differences were less evident following the alcohol-free beer intervention.

**Figure 6:**
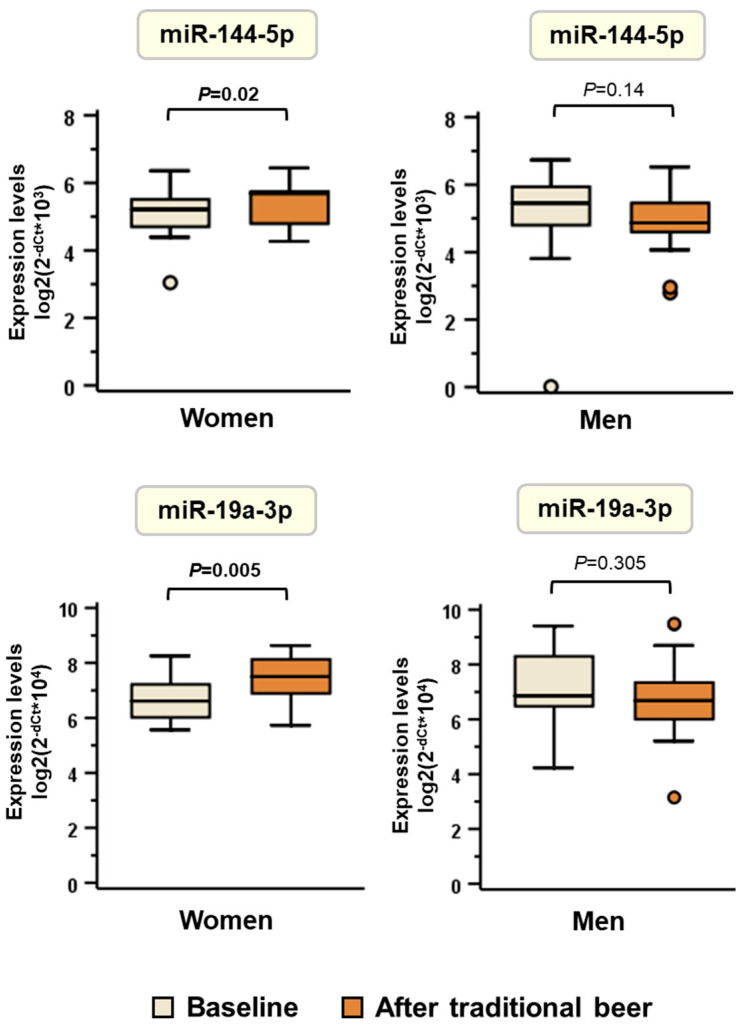
Expression levels of miR-19a-3p and miR-144-5p at baseline and after traditional beer consumption in women and men. Results are shown as medians (interquartile range, IQR: Q1–Q3). Box plots show the differential response between men (*n* = 21) and women (*n* = 15) after traditional beer compared to baseline status. Statistical significance was calculated by a paired Wilcoxon test.

Consistent with these observations, median [IQR] expression levels of miR-144-5p and miR-19a-3p significantly increased in women after the 4-week traditional beer intervention, whereas men showed a nonsignificant trend toward decreased expression (Figure 6). miR-144-5p and miR-19a-3p did not show significant changes relative to baseline after the consumption of alcohol-free beer. No changes were observed for the other three miRNAs (miR-1227-3p, miR-29c-3p, and miR-7-5p) either in men or in women after traditional or after alcohol free beer (Table S3). Therefore, these two miRNAs were selected for further analysis.

To investigate the baseline BMI-influenced miRNA responses to changes after beer consumption, we performed a comparative analysis between participants classified as overweight (BMI < 30 kg/m^2^; men = 11, women = 8) and those with obesity (BMI ≥ 30 kg/m^2^; men = 10, women = 7), with no significant differences in sex distribution between groups (Chi-square, *p* > 0.05). No significant changes in the five analysed miRNAs were observed after alcohol-free beer consumption. However, following traditional beer intake, the median change in miR-144-5p differed between participants with obesity and those with overweight, with a more negative shift observed in individuals with obesity compared with those with overweight [−0.49 (IQR −1.28 to 1.88) vs. 0.12 (IQR −0.189 to 0.978), *p* = 0.035]. A similar pattern was observed for miR-19a-3p, with participants with obesity showing a more negative median change compared with those with overweight [−0.62 (IQR −1.29 to 0.43) vs. 0.98 (IQR −0.03 to 1.14), *p* = 0.002]. Given the wide and overlapping interquartile ranges, these findings should be interpreted as exploratory trends rather than definitive BMI-dependent effects. Furthermore, when considering total miRNA expression levels, BMI was not associated with miR-144-5p or miR-19a-3p expression, either at baseline or after beer consumption.

### 3.4. Bioinformatics and System Biology Analysis: miR-144-5p and miR-19a-3p and Their Target Genes

In silico analyses were conducted to identify the tissue sources of the miR-144-5p and miR-19a-3p, their predicted target genes, and the signaling pathways potentially involved. The bioinformatic tools miTED, miRWalk, STRING (via Cytoscape), and KEGG-PANTHER (via ShinyGO) were used for these analyses.

**Figure 7:**
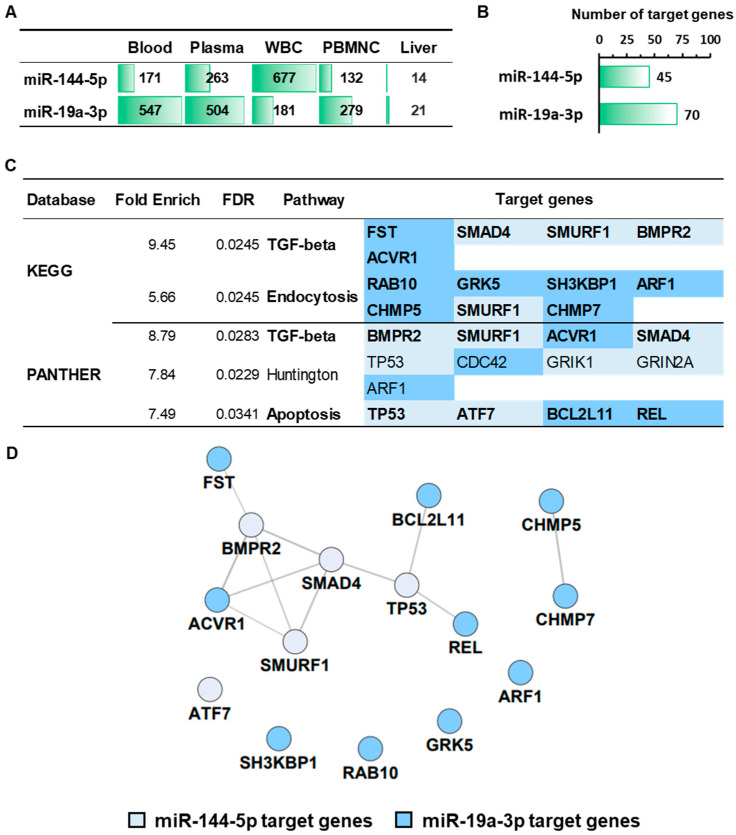
In silico analysis of miRNAs and their target genes associated with beer consumption. (**A**) Tissue sources of miR-144-5p and miR-19a-3p were obtained using the miTED database. WBC: white blood cells. PBMNC: peripheral blood mononuclear cells. (**B**) Number of target genes (protein-coding RNA) obtained from miRWalk and the Cytoscape platform. (**C**) The target genes were analyzed using the ShinyGO database (KEGG and PANTHER) to obtain the signaling pathways involved with the miRNA regulation. (**D**) In silico analysis by Cytoscape showed the target genes with molecular interactions.

To infer the tissue origins of the miRNAs in compartments relevant to whole blood and alcohol metabolism, we examined expression data using the miTED database, which aggregates Sequence Read Archive datasets and reports expression as Reads Per Million across healthy and diseased samples. In this resource, miR-144-5p showed the highest abundance in white blood cells, and miR-19a-3p showed greater concentrations in whole blood (Figure 7A).

Potential target genes for these miRNAs were predicted using miRWalk, selecting only targets with a binding score of 1 at the 3′UTR. Protein-coding RNA targets were then identified through STRING Protein Query accessed via Cytoscape. A total of 45 predicted target genes were found for miR-144-5p and 70 for miR-19a-3p regulation (Figure 7B).

Functional enrichment analysis of these target genes was performed using the ShinyGO database, classifying them according to the top KEGG and PANTHER signaling pathways. As shown in Figure 7C, in total, 18 target genes were significantly associated with two KEGG pathways and three PANTHER pathways. The significant KEGG pathways were GF-β signaling and endocytosis, which included SMAD4, SMURF1, and BMPR2 as predicted targets of miR-144-5p, and FST, ACVR1, RAB10, GRK5, SH3KBP1, ARF1, CHMP5, and CHMP7 as targets regulated by miR-19a-3p.

In the PANTHER pathway classification, TGF-β signaling also appeared, including ACVR1 (a direct target of miR-19a-3p), along with apoptosis signaling involving predicted targets for both miRNAs [miR-144-5p (TP53 and ATF7) and miR-19a-3p (BCL2L11 and REL)]. Overall, these 15 targets were more strongly associated with inflammation-related signaling pathways than with other categories, such as Huntington disease. Furthermore, the 15 target genes were analyzed by STRING Protein Query (Homo sapiens and confidence score: 0.7). This analysis revealed that 8 of the 15 genes formed a molecular interaction network, comprising four predicted targets of each miRNA (Figure 7D).

### 3.5. Bioinformatics and System Biology Analysis: miR-144-5p and miR-19a-3p and the Inflammasome Pathway

We previously demonstrated that beer consumption is associated with reduced expression of genes related to the inflammasome pathway [11].

**Figure 8:**
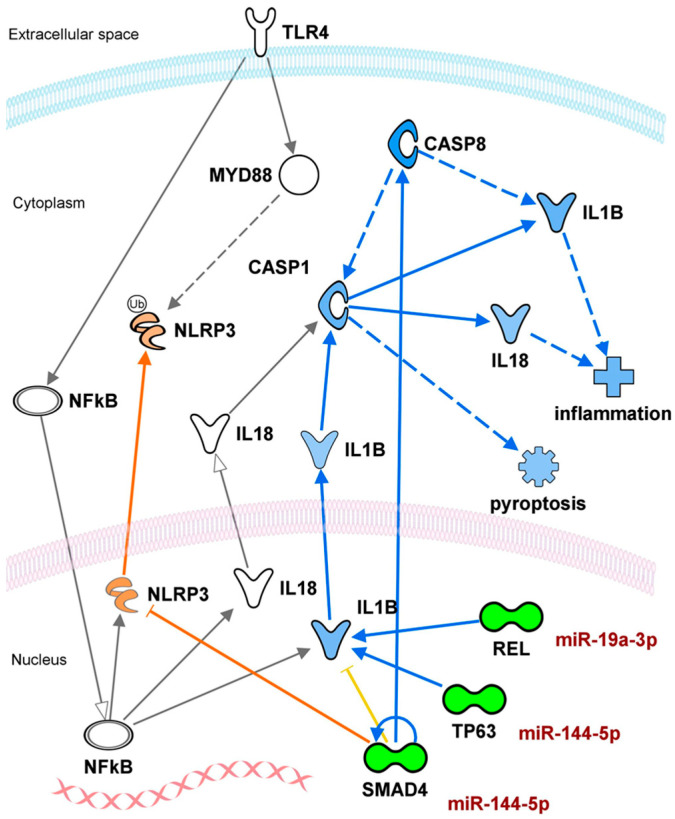
Relationship between miR-144-5p and miR-19a-3p and the inflammasome pathway. Molecular interaction between the miRNAs and their targets with the inflammasome obtained from IPA software. The direct targets for miR-144-5p and miR-19a-3p are indicated in green color. Predicted gene regulation is shown in orange (upregulation) or blue colors (downregulation) by MAP analysis. The green color refers to the effect of the miRNAs on their target genes. Molecules are shown according to their cell distribution. Direct and indirect molecular interactions are displayed as whole lines and dotted lines. Gray and yellow lines refer to low predictive power and inconsistent findings, respectively.

Based on the KEGG and PANTHER pathway analyses, eight inflammation-related target genes for miR-144-5p and miR-19a-3p were identified: these included transcription factors (SMAD4, TP53, and REL), kinases (BMPR2 and ACVR1), a microtubule binding (BCL2L11), a signal transducer activity (FST), and an enzyme (SMURF1). These genes, previously classified within the most relevant enriched pathways, were further analyzed in the context of the inflammasome using the Molecule Activity Predictor (MAP), a tool from IPA software (Figures S2 and Figure 8).

The molecular network showed that SMAD4, TP53, and REL have direct interactions with key inflammatory mediators (IL1B and IL18) and apoptosis/cell-survival molecules (CASP1 and CASP8), which are downstream targets of miR-144-5p (SMAD4, TP53) and miR-19a-3p (REL). These molecular interactions were mainly focused on IL1B, IL18, CASP1, and CASP8 and were not linked to the inflammasome complexes formed by NLRP3, NLRP1, AIM2, and NAIP (Figure S2).

Predicted downregulation of TP53 and REL inhibits IL1B in the nucleus and reduces CASP1 and IL1B in the cytoplasm. In addition, SMAD4 enhances the inhibitory effect on IL1B through CASP8 signaling (See Figures S2 and Figure 8). These results, as an exploratory study, show that an inhibition or reduction in cytokines is the predicted upstream effect caused by the downregulation of target genes for miR-19a-3p and miR-144-5p. Thus, there is a high probability that cytokine inflammation will be reduced through the downregulation of target genes caused, according to predictions, by the upregulation of the miRNAs.

## 4. Discussion

Moderate alcohol consumption in the form of fermented beverages is inversely correlated with adverse cardiovascular outcomes, conferring protective effects against cardiovascular disease. However, excessive alcohol intake is associated with high cardiovascular risk [21]. Low to moderate doses of alcohol are related to decreased inflammatory markers [22], while high doses promote oxidative stress and the activation of inflammatory signaling [23]. Previous studies performed with beer showed that the alcohol fraction of traditional beer reduced plasma inflammatory biomarkers related to atherosclerosis, while the polyphenol content reduced leukocyte activation [24] and can modulate inflammatory response in macrophages [25], but whether beer consumption could induce epigenetic changes through miRNA modification was not known. Thus, we provide for the first time in the whole blood of healthy subjects the differential miRNA profile induced by consuming moderate amounts of alcohol-free beer and traditional beer with meals. Our results also show that these differential levels are associated with sex differences following beer consumption. Exploratory sex-stratified analyses suggested a tendency toward higher post-intervention levels in women compared with men after traditional beer consumption, whereas no clear sex-related differences were observed following alcohol-free beer intake. The observed miRNA changes are consistent with the moderate and regular intake of beer in an otherwise healthy overweight/obese population, supporting their physiological relevance in the context of dietary exposure.

Some metabolic differences between sexes may help to explain the differences observed in our study. Body fat percentage is greater in women than in men; women also have a lower percentage of water in their bodies and a reduced activity of alcohol dehydrogenase, leading to higher blood alcohol concentrations than men [9,26]. Due to these characteristics, women are less efficient than men in their metabolization of alcohol, suggesting that women are more vulnerable to alcohol-induced liver damage than men. In our study, women consumed half as much beer as men, and yet their levels of miR-144-5p and miR-19a-3p were higher. Women in the study were healthy with non-pathological concentrations of ASAT and GGT.

Despite the limited information on miRNAs associated with beer consumption in humans, previous studies in animal models exposed to chronic high-dose alcohol have reported differential profiles of miR-98-5p and miR-3541 in males, associated with inflammatory signaling (cytokines and Wnt pathway), and miR-500-3p and miR-20b-5p in females, associated with Wnt signaling and the TGF-beta signaling pathway [27].

In the present study, moderate daily beer intake (with standardized doses for men and women) was associated with changes in miR-144-5p and miR-19a-3p expression, miRNAs whose predicted target genes are involved in pathways related to TGF-β signaling, apoptosis, and immune regulation, including TP53, SMAD4, and REL [28,29,30].

Low expression of miR-19a-3p has been reported in uncontrolled cell migration, whereas its upregulation suppresses invasion and cell migration through downstream effectors of TGF signaling [28]. Inflammatory signaling linked to IL1B can mediate TGF-beta expression and contribute to the activation of the inflammasome complex (NLRP3) [31], suggesting a potential mechanistic link between TGF-β–related pathways and inflammasome regulation. In this context, epigenetic modulation of miR-19a-3p and miR-144-5p may be relevant to pathways involved in inflammatory regulation. In addition, high levels of miR-144-5p have been shown to inhibit Ang II signaling in an Apoe^−/−^ mouse model of abdominal aortic aneurysm, promoting an anti-inflammatory polarization of macrophages and improved survival [32]. Taken together, the miRNA changes observed in the present study are consistent with pathways previously linked to inflammatory regulation and may contribute to a mechanistic framework connecting moderate beer consumption to immune-related signaling, particularly in women. In line with this interpretation, we previously reported that serum collected after the same beer intervention attenuated inflammasome activation in macrophages exposed to inflammatory stressors [11]. Thus, although direct measurements of cytokines or inflammasome components were not performed in this study, the present findings suggest that miR-144-5p and miR-19a-3p could represent candidate epigenetic mediators linking moderate beer consumption to immune-related pathways; however, direct mechanistic studies will be required to confirm their role in inflammasome regulation.

The current study has some limitations. Although it is based on a well-characterized cohort of healthy but overweight/obese individuals and uses a longitudinal cross-over design that strengthens within-subject comparisons, the sample size is relatively small. In addition, as expected for an exploratory discovery screen conducted in a limited subset of participants, the microarray analysis was not powered for formal multiple-testing correction and was therefore used to prioritize candidate miRNAs for subsequent targeted quantitative validation. Moreover, miRNA profiling was performed in whole blood collected at a single study site; therefore, the findings primarily reflect systemic blood-derived miRNA responses and may not be directly extrapolated to tissue-specific effects or to populations with different clinical characteristics.

## 5. Conclusions

In summary, this exploratory secondary analysis shows that moderate and regular consumption of traditional beer with meals is associated with changes in whole-blood miRNA expression in an otherwise healthy overweight/obese population. Specifically, miR-144-5p and miR-19a-3p emerged as candidate miRNAs whose expression patterns are consistent with pathways involved in immune and inflammatory regulation. Although these findings do not establish causality or direct mechanistic effects, they provide novel evidence that moderate beer consumption may be linked to physiologically relevant epigenetic modulation. Future studies incorporating direct measurements of cytokines, inflammasome components, and functional validation will be required to confirm the mechanistic role of these miRNAs.

## Data Availability

The raw data supporting the conclusions of this article will be made available by the authors on request. Whole blood miRNA data obtained by the Affymetrix microarrays were deposited in NCBI’s Gene Expression Omnibus and are accessible under the accession number GSE310134 (https://www.ncbi.nlm.nih.gov/geo/query/acc.cgi?acc=GSE310134, accessed on 15 September 2025). The information outlined in the manuscript, as well as the code book and analytic code, will be provided upon a reasonable request, subject to scientific approval.

## Supplementary Materials

The following supporting information can be downloaded at: https://www.mdpi.com/article/10.3390/nu18010149/s1, Figure S1: Quality of real-time PCR reaction in whole blood RNA of healthy subjects, Figure S2: Relationship between target genes of miR-144-5p and miR-19a-3p with canonical pathway of inflammasomes, Table S1: Clinical characteristics of healthy subjects at baseline status, Table S2: List of assays, Table S3: miRNA expression levels in whole blood of men and women at baseline and after 4 weeks intervention with alcohol-free beer and traditional beer.
