# Supplementary material for: miRNA Expression Profile in Whole Blood of Healthy Volunteers and Moderate Beer Consumption with Meals
Source: Nutrients. 2026 Jan 1;18(1):149. doi: 10.3390/nu18010149 (PMC12787979; doi:10.3390/nu18010149)
Supplement: Supplementary file 1 [file nutrients-18-00149-s001.zip › nutrients-4041072-supplementary.pdf]

miRNA expression profile in whole blood of healthy volunteers and moderate beer consumption with meals

Teresa Padro[1,2*,†], Rafael Escate[1,2†], Lina Badimon [1,2,3,4]

[1] Institut Recerca Sant Pau (IR Sant Pau), Sant Quintí, 77-79, 08041 Barcelona, Spain; rescate@santpau.cat (R.E.); lbadimon@ficsi.org (L.B.)

[2] Centro de Investigación Biomédica en Red Cardiovascular (CIBERCV), Instituto de Salud Carlos III, 28029 Madrid, Spain

[3] Medical School, Universitat de VIC-UCC and IRIS-CC, 08500 Vic, Barcelona, Spain

[4] Cardiovascular Research Foundation for Health Prevention and Innovation (FICSI), 08017 Barcelona, Spain

† These authors contributed equally to this work.

*** Correspondence:**

Dr. Teresa Padro

Institut Recerca Sant Pau (IR-HSCSP)

Sant Quintí, 77-79 - 08041 Barcelona

Tel: (+34) 935565886

E-mail: tpadro@santpau.cat

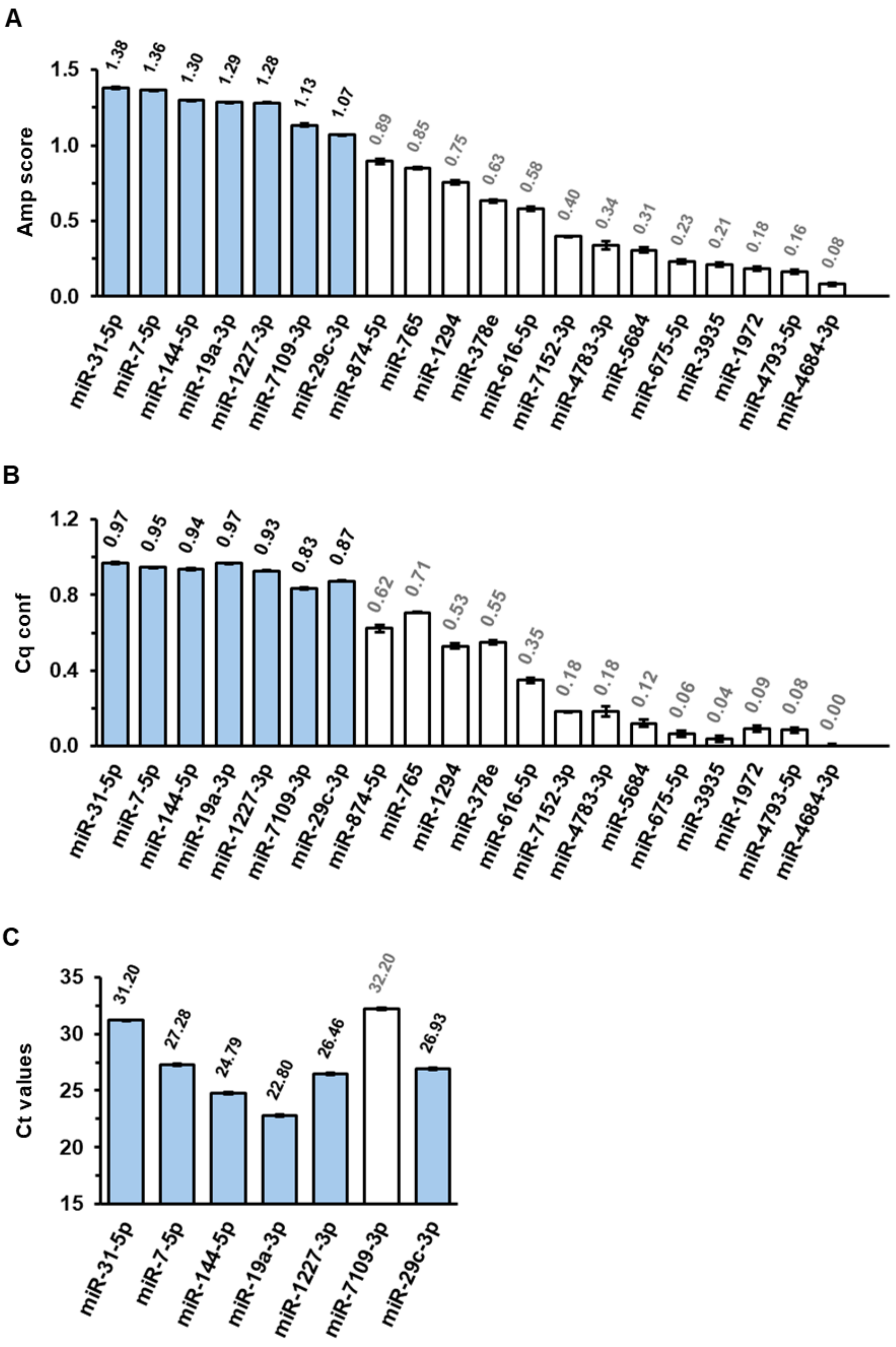

Figure S1. Quality of real-time PCR reaction in whole blood RNA of healthy subjects microRNA (miRNA) amplifications were assessed by quality parameters such as **(A)** amplification score (Amp Score: > 1), **(B)** Cq confidence score (Cq Conf > 0.8) and **(C)** Ct values (Ct <32 cycles). The miRNAs displayed in light blue bar chart indicate suitable quality.

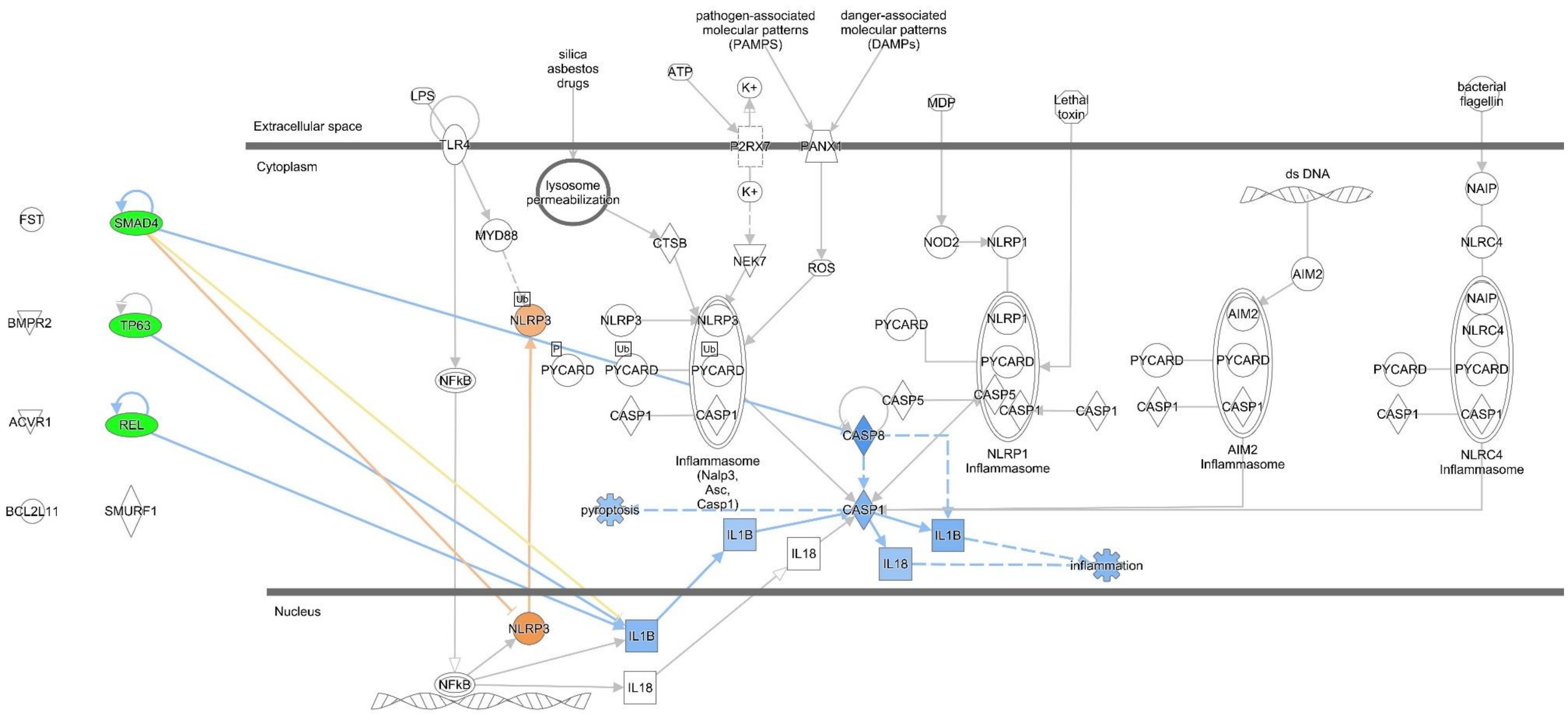

Figure S2. Relationship between target genes of miR-144-5p and miR-19a-3p with canonical pathway of inflammasome

The target genes are grouped on the left side. Inflammasome signalling pathway was obtained by using IPA software. Molecule activity predicter (MAP) tool from IPA evidenced that a downregulation of the target genes (green colour) leads to a decrease in inflammatory signalling in the inflammasome pathway. Molecules and interactions with predicted activation are shown in orange intensities and those with predicted inhibition are shown in blue intensities. Direct and indirect molecular interactions are displayed as whole line and dotted lines. Gray and yellow lines refer to low predictive power and inconsistent findings, respectively.

Table S1. Clinical characteristics of healthy subjects at baseline status

Anthropometric parameters	Affymetrix phase	Real time PCR phase
Male/Female, *n*	3/7	21/15
Age (years) of inclusion in cohort	50 (47 – 55)	48 (44 – 52)
Body Mass Index (BMI) (Kg/m^2)	31 (28 – 34)	29 (28 – 32)
**Biochemical parameters**		
Fasting plasma glucose (mg/dL)	84 (81 – 90)	87 (83 – 96)
Creatinine (mg/dL)	0.68 (0.66 – 0.74)	0.75 (0.68 – 0.84)
Urea (mg/dL)	13 (11 – 13)	13 (12 – 16)
ASAT (U/L)	16 (14 – 17)	16 (15 – 19)
GGT (U/L)	13 (12 – 16)	19 (13 – 24)
**Lipid parameters**		
Total cholesterol (mg/dL)	185 (167 – 213)	183 (167 – 209)
LDL-cholesterol (mg/dL)	121 (106 – 139)	123 (105 – 141)
HDL-cholesterol (mg/dL)	50 (45 – 56)	47 (42 – 52)
VLDL-cholesterol (mg/dL)	17 (10 – 20)	16 (11 – 20)
Non-HDL (mg/dL)	133 (119 – 163)	134 (120 – 166)
Triglycerides (mg/dL)	83 (51 – 98)	81 (57 – 99)
**Haematological parameters**		
Red cell blood (10^6 mm)	4.35 (4.19 – 4.62)	4.37 (3.97 – 4.67)
White cell blood (10^3 mm^3)	5.8 (5.2 – 6.9)	5.75 (5.2 – 6.25)
Haematocrit (%)	37 (33 – 38)	37 (34 – 40)
Platelet (10^3 mm^3)	216 (193 – 235)	205 (169 – 222)

Clinical data are represented as medians (Interquartile range, IQR: Q1 – Q3) or number of cases (*n*). ASAT, Aspartate Aminotransferase; GGT, Gamma-Glutamyl Transferase; LDL, low-density lipoprotein; HDL, high-density lipoprotein; VLDL: very low-density lipoprotein.

Table S2. List of assays

miRNAs	Assay ID	Accession
**hsa-miR-765**	479173_mir	MIMAT0003945
**hsa-miR-1972**	478746_mir	MIMAT0009447
**hsa-miR-29c-3p**	479229_mir	MIMAT0000681
**hsa-miR-7-5p**	483061_mir	MIMAT0000252
**hsa-miR-675-5p**	478196_mir	MIMAT0004284
**hsa-miR-144-5p**	477914_mir	MIMAT0004600
**hsa-miR-5684**	480146_mir	MIMAT0022473
**hsa-miR-19a-3p**	479228_mir	MIMAT0000073
**hsa-miR-31-5p**	478015_mir	MIMAT0000089
**hsa-miR-1294**	478693_mir	MIMAT0005884
**hsa-miR-3935**	479754_mir	MIMAT0018350
**hsa-miR-7152-3p**	480558_mir	MIMAT0028215
**hsa-miR-4793-5p**	480054_mir	MIMAT0019965
**hsa-miR-874-5p**	479183_mir	MIMAT0026718
**hsa-miR-4684-3p**	479919_mir	MIMAT0019770
**hsa-miR-378e**	478537_mir	MIMAT0018927
**hsa-miR-4783-3p**	480044_mir	MIMAT0019947
**hsa-miR-7109-3p**	480544_mir	MIMAT0028116
**hsa-miR-1227-3p**	478642_mir	MIMAT0005580
**hsa-miR-616-5p**	479099_mir	MIMAT0003284
**hsa-miR-16-5p**	477860_mir	MIMAT0000069
**cel-miR-39**	478293_mir	MIMAT0000010

Assays used for miRNA analysis in whole blood samples by real-time PCR reaction using Taqman advanced technology.

Table S3. miRNA expression levels in whole blood of men and women at baseline and after 4 weeks intervention with alcohol-free beer and traditional beer

		**Baseline (1)**	**After alcohol-free beer (2)**	**After traditional beer (3)**	***P*-value (1 *vs* 2)**	***P*-value (1 *vs* 3)**
**Men**	**miR-144-5p**	5.45 (4.79 - 5.94)	5.12 (4.57 - 5.32)	4.87 (4.59 - 5.46)	0.434	0.14
	Low-IC	4.6	4.79	4.49		
	Up-IC	5.9	5.51	5.32		
	**miR-1227-3p**	3.34 (2.85 - 4.24)	3.59 (3.18 - 4.28)	3.13 (2.93 - 3.78)	0.520	0.23
	Low-IC	3	3.21	2.79		
	Up-IC	4.43	3.98	4.05		
	**miR-29c-3p**	3.02 (2.06 - 3.39)	2.82 (2.31 - 3.53)	2.66 (2.24 - 3.18)	0.986	0.375
	Low-IC	2.37	2.28	2.23		
	Up-IC	3.67	3.53	2.99		
	**miR-7-5p**	2.89 (2.57 - 3.36)	2.89 (2.59 - 3.02)	2.72 (2.44 - 3.10)	0.375	0.058
	Low-IC	2.69	2.61	2.51		
	Up-IC	3.38	3	3.14		
	**miR-19a-3p**	6.86 (6.47 - 8.29)	7.06 (5.95 - 8.06)	6.69 (6.01 - 7.34)	0.664	0.305
	Low-IC	6.47	6.05	6.11		
	Up-IC	7.74	7.53	7.46		
**Women**	**miR-144-5p**	**5.21 (4.7 - 5.51)**	5.29 (4.29 - 5.71)	**5.69 (4.79 - 5.74)**	0.691	**0.02**
	Low-IC	4.65	4.67	5.05		
	Up-IC	5.54	5.55	5.79		
	**miR-1227-3p**	3.73 (3.33 - 4.51)	3.45 (3.04 - 4.42)	3.44 (2.90 - 4.03)	0.955	0.112
	Low-IC	3.29	3.09	3.09		
	Up-IC	4.46	4.34	4.02		
	**miR-29c-3p**	2.93 (2.72 - 4.08)	3.62 (3.01 - 3.99)	3.51 (3.06 - 3.72)	0.307	0.691
	Low-IC	2.64	3.04	3.13		
	Up-IC	3.88	3.93	3.71		
	**miR-7-5p**	2.43 (2.12 - 3.06)	2.62 (2.35 - 2.88)	2.39 (2.23 - 2.79)	0.307	0.691
	Low-IC	2.07	2.3	2.25		
	Up-IC	2.89	3	2.83		
	**miR-19a-3p**	**6.61 (6.02 - 7.22)**	6.84 (6.29 - 7.54)	**7.50 (6.89 -8.13)**	0.460	**0.005**
	Low-IC	6.19	6.04	6.93		
	Up-IC	7.12	7.74	7.91		

miRNA levels after consumption of alcohol-free beer and traditional beer compared to baseline status. Results are shown as medians (Interquartile range, IQR: Q1 – Q3). Lower limit and upper limit of 95% confidence interval are also included (Low-IC and Up-IC). Statistical significance was calculated by paired-Wilcoxon test.
